# Outcomes after fertility‐sparing surgery of early‐stage ovarian cancer: A nationwide population‐based study

**DOI:** 10.1002/cam4.7132

**Published:** 2024-04-12

**Authors:** Chia‐Yi Lee, Chun‐Ju Chiang, Yi‐Jou Tai, Heng‐Cheng Hsu, Yu‐Li Chen, Ying‐Cheng Chiang, Chia‐Ying Wu, Wen‐Chung Lee, Hsiao‐Lin Hwa, Wen‐Fang Cheng

**Affiliations:** ^1^ Department of Obstetrics and Gynecology National Taiwan University Hospital, Hsin‐Chu Branch Hsinchu Taiwan; ^2^ Department of Obstetrics and Gynecology National Taiwan University Hospital Taipei Taiwan; ^3^ Graduate Institute of Epidemiology and Preventive Medicine, College of Public Health National Taiwan University Taipei Taiwan; ^4^ Taiwan Cancer Registry Taipei Taiwan; ^5^ Graduate Institute of Clinical Medicine, College of Medicine National Taiwan University Taipei Taiwan; ^6^ Department of Obstetrics and Gynecology National Taiwan University Hospital Douliou Taiwan; ^7^ Department of Obstetrics and Gynecology Nantou Hospital of the Ministry of Health and Welfare Nantou City Taiwan; ^8^ Department and Graduate Institute of Forensic Medicine, College of Medicine National Taiwan University Taipei Taiwan; ^9^ Graduate Institute of Oncology, College of Medicine National Taiwan University Taipei Taiwan

**Keywords:** fertility‐sparing surgery, outcome, ovarian cancer, population study, staging surgery, survival

## Abstract

**Background:**

Fertility‐sparing surgery (FSS) is an alternative choice of young patients who have not completed their family planning and still have fertility needs. The aims of this study were to compare the outcomes of early‐stage epithelial ovarian cancer (EOC) patients undergoing FSS and radical comprehensive staging surgery (RCS), and the suitability of FSS.

**Methods:**

A total of 1297 patients aged between 20 and 44 years with newly diagnosed early‐stage EOC were recruited from the Taiwan Cancer Registry database between 2009 and 2017. Site‐specific surgery codes were used to distinguish patients in FSS group or RCS group. Cancer‐specific survival (CSS) was evaluated using Kaplan–Meier method with log‐rank test and Cox regression model.

**Results:**

There were 401 and 896 patients in FSS and RCS group. Patients in FSS group were with younger age and mostly had Stage I disease. In contrast, patients in RCS group were older. There were more Stage II, high‐grade (Grade 3) disease, and adjuvant chemotherapy in RCS group. Stage and tumor grade were two independent factors correlating with CSS and the type of surgery showed no effect on CSS (HR: 1.09, 95% CI: 0.66–1.77, *p* = 0.73) in multivariable analysis. In multivariable analysis, the clear cell carcinoma group who underwent FSS demonstrated better CSS compared to those in the RCS group (HR: 0.28, 95% CI: 0.06–0.82, *p* = 0.04). A total of 17 women who underwent FSS developed second malignancies of the uterine corpus or contralateral ovary.

**Conclusion:**

FSS can be a safe alternative procedure in selected young patients of Stage I EOC who have fertility desire. Endometrial biopsy before or during FSS and regular surveillance to detect recurrence are mandatory for ovarian cancer patients undergoing FSS.

## Graphical abstract

Fertility‐sparing surgery (FSS) can be a safe alternative procedure in selected young patients of early‐stage epithelial ovarian cancer who have fertility desire. Endometrial biopsy should be performed before or during FSS. Regular surveillance to detect recurrence is mandatory for ovarian cancer patients undergoing FSS.

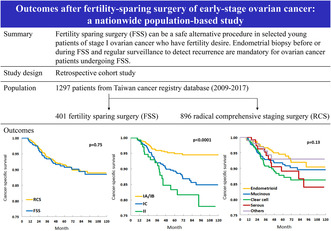

## 1 INTRODUCTION

Ovarian cancer affects women of all ages, and is the ninth most common newly diagnosed malignancy among women worldwide.^1^ In 2020, there were 31,4000 new cases of ovarian cancer, and 20,7000 deaths from ovarian cancer globally.^1^ In Taiwan, there are approximately 1677 new cases of ovarian cancer and 683 deaths from ovarian cancer annually,^2^ and the incidence rate keeps raising.^3^ At the time of ovarian cancer diagnosis, 23.1% of patients are between 20 and 45 years of age.^2^ These patients are still of reproductive age when diagnosed, and may not have completed their family planning.^4^ Loss of fertility due to malignancy treatment can result in grief, stress, sexual dysfunction, and depression among patients of reproductive age.^5^ Therefore, treatments that preserve fertility—especially without compromising oncologic outcomes—are important for these young patients.

For early‐stage epithelial ovarian cancer (EOC), the standard surgical treatment has traditionally been total hysterectomy, bilateral salpingo‐oophorectomy (BSO), plus peritoneal and lymph‐node sampling.^6^ In Taiwan, the complete staging surgery also includes omentectomy and ascites cytology or peritoneal wash cytology examination.^7^

In Taiwan, we follow the Clinical Practice Guideline of Gynecologic Oncology proposing by Taiwan Cooperative Oncology Group (TGOG) for patient selection^7^ to treat our patients. According to this guideline, the standard management of early‐stage EOC is a radical comprehensive staging surgery (RCS) including total hysterectomy, BSO, omentectomy, retroperitoneal lymph node dissection, peritoneal biopsy, and ascites cytology or peritoneal wash cytology examination. The candidates of FSS are those who have fertility desire and whose tumor grossly confined to ovaries under surgical inspection.

Reproductive‐age patients with early‐stage disease may have the option of fertility‐sparing surgery (FSS), although the recommended indications remain controversial.^6^ According to the European society for medical oncology (ESMO) clinical practice guidelines, FSS can be considered for patients with Stage IA or IC disease, favorable histology (non‐clear cell, i.e., mucinous, serous, endometrioid, or mixed histology), and Grade 1 or 2 disease.^8^ Additionally, patients with Stage IA clear cell carcinoma are considered acceptable candidates according to Satoh et al.^6^ and the Asian society of gynecologic oncology (ASGO) international workshop 2014.^9^ Moreover, the NCCN clinical practice guidelines state that patients with Stage IB disease who desired FSS can receive BSO to preserve the uterus.^10^ Limited available evidence shows that FSS can be a safe procedure for selected young women with early‐stage EOC.^7^ After surgery, adjuvant chemotherapy is mandatory to most patients. The investigation of immunotherapy in early‐stage EOC and the identification of suitable patients are currently underway.

Due to the difficulty of designing and performing prospective randomized clinical trials, it has not yet been clearly demonstrated whether patients with early‐stage EOC and hope for future reproduction can safely undergo FSS rather than RCS. Investigations of this subject have mainly been retrospective with limited patient numbers.^11^, ^12^, ^13^, ^14^, ^15^, ^16^, ^17^, ^18^ In the present study, we aimed to survey the oncologic outcomes of early‐stage EOC patients who underwent FSS or RCS, and to analyze factors influencing their oncologic outcomes. Based on our findings, we propose selective criteria for FSS in cases of early‐stage EOC.

## 2 MATERIALS AND METHODS

### 2.1 Study design and data source

We conducted a retrospective study using data from the nationwide Taiwan Cancer Registry (TCR) database. This study was reviewed and approved by the Institutional Review Board of the National Taiwan University Hospital. The TCR is one of the highest quality cancer registries in the world, and records clinical data, such as cancer staging, laboratory values, and detailed treatment information for patients with newly diagnosed malignancies in Taiwan.^19^

**FIGURE 1:**
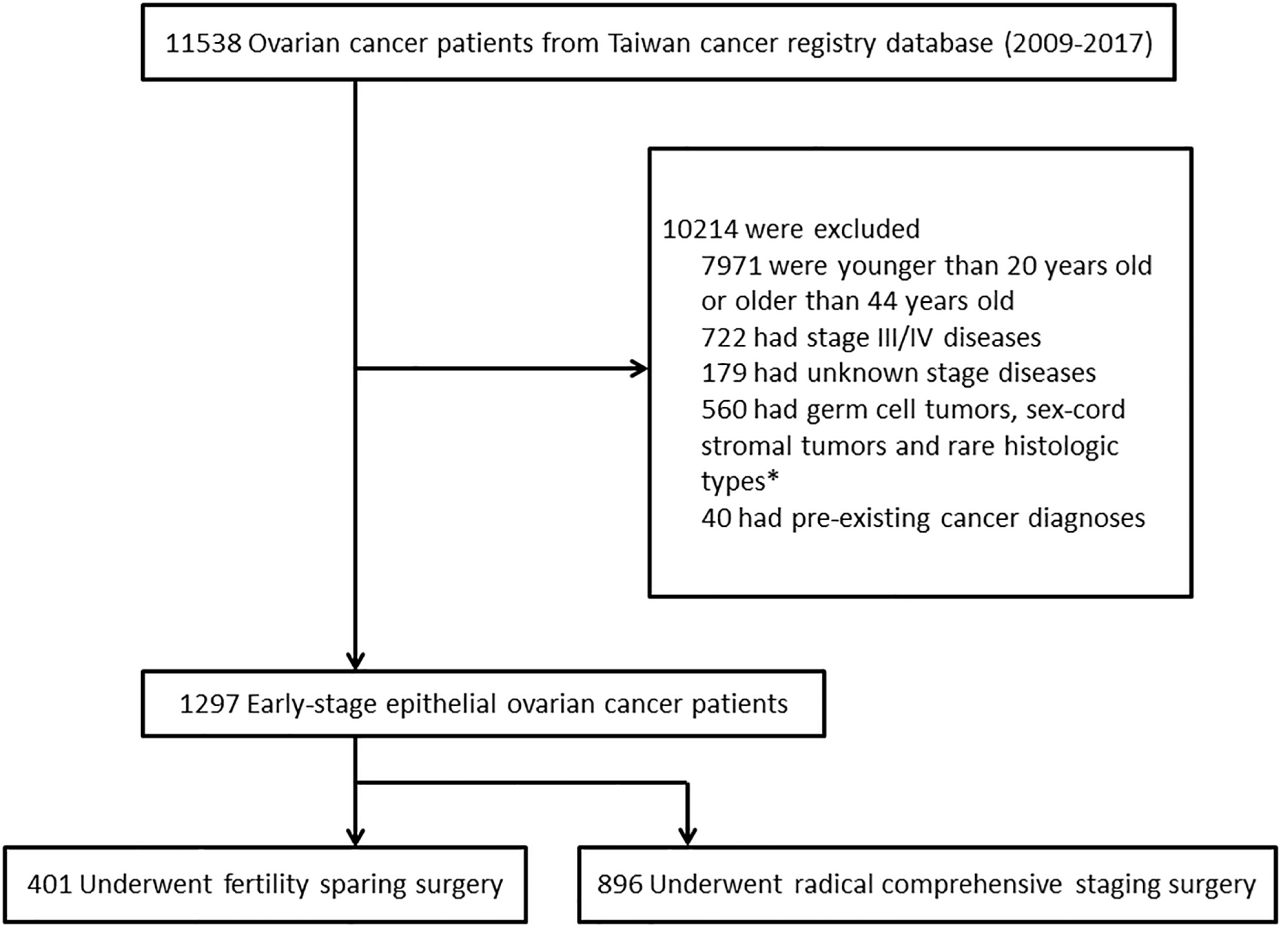
Flowchart of patient selection in this study. *Rare histologic types including large cell neuroendocrine carcinoma, small cell carcinoma, and carcinosarcoma were excluded.

The flowchart of patient selection in this study is shown in Figure 1. We retrieved information regarding patients who were newly diagnosed with ovarian cancer (ICD‐O‐3, code: C56) from 2009 to 2017. Women between 20 and 44 years of age were considered fertile and potentially in need of fertility preservation. FSS candidates were patients with early‐stage EOC—for example, Stages I and II, according to the American Joint Committee on Cancer (AJCC) staging guidelines (6th edition in 2009, and 7th edition in 2010–2017). The four main histological types of EOC were serous, mucinous, endometrioid, and clear cell carcinomas. This study also included mixed cell adenocarcinoma and unspecified types of carcinoma/adenocarcinoma. Patients were excluded if they had been diagnosed with other malignancies more than 3 months prior to their EOC diagnosis, to avoid the possibility of metastatic ovarian cancer. For included patients, we collected the following data: age at diagnosis, year of diagnosis, histology, cancer stage, tumor grade, surgical types, and adjuvant chemotherapy. The low‐grade serous ovarian carcinoma was defined as Grade 1 carcinoma and clear cell carcinoma was defined as Grade 3 carcinoma.

Patients who underwent unilateral salpingo‐oophorectomy (USO) or BSO without hysterectomy were regarded as undergoing FSS, regardless of whether they received total, partial, or no omentectomy. Patients with surgery codes, such as USO or BSO and hysterectomy with or without omentectomy, debulking surgery, or pelvic exenteration, were classified into the RCS group. Adjuvant chemotherapy was recommended for ovarian cancer patients except those with Stage IA, Grade 1 diseases. The chemotherapeutic regimen was platinum (carboplatin (AUC 5‐7.5) or cisplatin (75 mg/m^2^)) with paclitaxel (175 mg/m^2^) or cyclophosphamide (600 mg/m^2^) for three to six cycles depending on the doctors' and patients' choice.

### 2.2 Statistical analysis

Descriptive analysis was used to present the patients' basic characteristics, including age at diagnosis, histology, stage, grade, adjuvant chemotherapy, and second malignancy. The age difference between groups was analyzed by one‐way ANOVA, and between‐group differences in other categorical parameters were analyzed by chi‐squared test. The main outcome measure was cancer‐specific survival (CSS), which was defined as the length of time from either the date of diagnosis or the start of treatment for cancer, to the date of death from the disease. All cases were followed up through data linkage to the Death Registration Database until December 31, 2018. Cumulative CSS plots were generated using the Kaplan–Meier method. CSS was compared between the two groups using the log‐rank test. Univariate and multivariable Cox proportional hazards models were used to evaluate the factors including age, histology, stage, grade, type of surgery, and adjuvant chemotherapy which are important parameters that might influence the outcome of ovarian cancer patients. A *p* value of <0.05 was interpreted as indicating statistical significance. All analyses were performed using SAS software, version 9.4 (SAS Institute Inc., Cary, NC).

## 3 RESULTS

### 3.1 Basic characteristics of 1297 early‐stage EOC patients

This study included a total of 1297 early‐stage EOC patients: 401 in the FSS group and 896 in the RCS group. Table 1 presents the clinico‐pathologic characteristics of the FSS and RCS groups. The mean age at diagnosis was 32.4 ± 5.9 years for the FSS group, and 38.8 ± 4.7 years for the RCS group. The median follow‐up time were 57.1 months (4.5–119.6 months) and 57.0 months (5.7–118.8 months) in FSS and RCS groups. Patients in RCS group were significantly older than patients in the FSS group (*p* < 0.001). In the FSS group, the most common histologic type was mucinous carcinoma (167 out of 401, 41.5%). In the RCS group, the most common histologic types were endometrioid carcinoma (289 out of 896, 32.3%), followed by clear cell carcinoma (259 out of 896, 28.9%). The majority of patients had Stage I disease. Stage II disease was diagnosed in 5.2% (21 out of 401) of patients in the FSS group, and 13.7% (123 out of 896) patients of the RCS group (*p* < 0.001, chi‐squared test). In the FSS group, there were more low‐grade tumors (Grades 1 and 2, 184 out of 401, 45.9%) than high‐grade tumors (Grade 3, 109 out of 401, 27.2%). In the RCS group, the proportions of low‐grade and high‐grade tumors were 44.2% (396 out of 896) and 42.6% (382 out of 896) respectively. The percentage of high‐grade tumors was higher in the RCS group than in the FSS group (*p* < 0.001, chi‐squared test). Adjuvant chemotherapy was more common in the RCS group than in the FSS group: 77.9% (698 out of 896) versus 53.6% (215 out of 401) (*p* < 0.001) **(**Table 1 **)**. The basic characteristics of 1297 early‐stage EOC patients undergoing adjuvant chemotherapy or not are shown in Table S1. Patients who did not receive chemotherapy were younger and had a higher proportion of mucinous histology, Stage IA/IB diseases, and Grade 1/2 tumors compared to those who did receive chemotherapy. However, patients who underwent adjuvant chemotherapy had higher proportion of undergoing RCS than those without adjuvant chemotherapy.

**TABLE 1:** Basic characteristics of 1297 patients with early‐stage EOC treated with FSS or RCS.

Characteristics	FSS (*N* = 401) (%)	RCS (*N* = 896) (%)	*p*
Age, mean ± SD	32.4 ± 5.9	38.8 ± 4.7	**<0.001**
Age			
<35 years	249 (62.1)	157 (17.5)	**<0.001**
35–39 years	107 (26.7)	253 (28.3)	
≥40 years	45 (11.2)	486 (45.2)	
Histology			
Serous carcinoma	42 (10.5)	81 (9.0)	**<0.001**
Mucinous carcinoma	167 (41.5)	198 (22.1)	
Endometrioid carcinoma	75 (21.5)	289 (32.3)	
Clear cell carcinoma	70 (17.5)	259 (28.9)	
Others^a^	36 (9.0)	69 (7.7)	
Stage			
IA + IB	212 (52.9)	365 (40.8)	**<0.001**
IC	168 (41.9)	408 (45.5)	
II	21 (5.2)	123 (13.7)	
Grade			
1 + 2	184 (45.9)	396 (44.2)	**<0.001**
3	109 (27.2)	382 (42.6)	
Unknown	108 (26.9)	118 (13.2)	
Adjuvant chemotherapy			
No	186 (46.4)	198 (22.1)	**<0.001**
Yes	215 (53.6)	698 (77.9)	

Abbreviations: EOC, epithelial ovarian cancer; FSS, fertility‐sparing surgery; *N*, number of patients; NOS, not otherwise specified; RCS, radical comprehensive staging surgery, SD, standard deviation.

^a^ Others included mixed cell adenocarcinoma; adenocarcinoma, NOS; and carcinoma, NOS.

### 3.2 Stage and tumor grade influenced the CSS of early‐stage EOC patient

**FIGURE 2:**
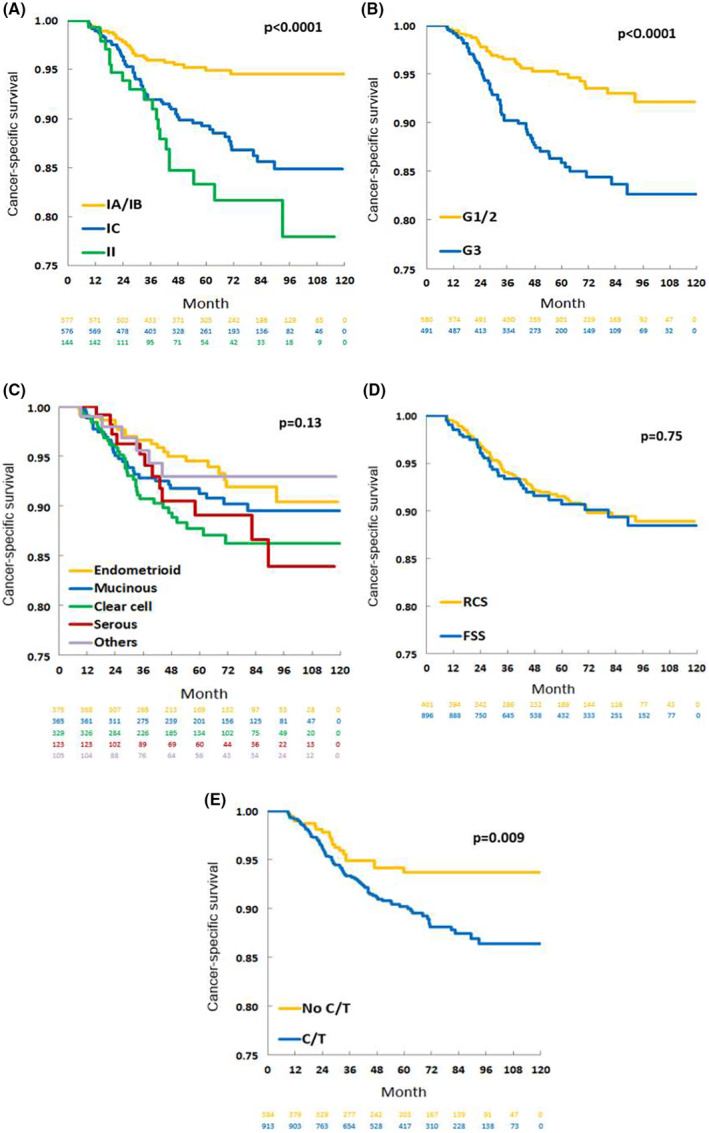
Kaplan–Meier analyses of cancer‐specific survival among 1297 patients with early‐stage epithelial ovarian cancer stratified according to different clinico‐pathologic characteristics: (A) stage, (B) tumor grade, (C) histologic type, (D) surgical procedure, and (E) adjuvant chemotherapy. C/T, chemotherapy; EOC, epithelial ovarian cancer; G, grade; FSS, fertility‐sparing surgery; RCS, radical comprehensive staging surgery.

CSS was analyzed with stratification according to clinico‐pathologic factors. As shown in Figure 2A, CSS was associated with disease stage, with Stage IA/IB diseases showing better CSS than Stage IC/II diseases (*p* < 0.0001, Figure 2A **)**. CSS did not significantly differ between Stage IC and Stage II diseases (*p* = 0.54, log‐rank test). Additionally, CSS was better in women with Grade 1/2 tumors than those with Grade 3 tumors (*p* < 0.0001, Figure 2B). CSS did not significantly differ among different histologic types (*p* = 0.13, Figure 2C).

### 3.3 Adjuvant chemotherapy, regardless of surgical procedure, correlated with CSS among early‐stage EOC patients

We further evaluated whether surgical procedure or adjuvant chemotherapy influenced outcome among the total analyzed group of 1297 patients with early‐stage EOC. CSS was similar between women who underwent FSS and those who underwent RCS (*p* = 0.75, Figure 2D). CSS was significantly worse in patients who received adjuvant chemotherapy than in those who did not (*p* = 0.009, Figure 2E).

### 3.4 Stage and tumor grade were two independent risk factors affecting the outcome of early‐stage EOC patients

Multivariable analysis was performed to analyze factors that were correlated with CSS among the 1297 early‐stage EOC patients. As shown in Table 2, the independent poor prognostic factors for CSS included stage (Stage IC, HR 2.40, 95% CI 1.47–4.05, *p* < 0.001; Stage II, HR 3.60, 95% CI 1.86–6.93, *p* < 0.001) and histologic grade (Grade 3, HR 3.19, 95% CI 1.79–5.65, *p* < 0.001). After adjustment, the other tested variables did not significantly affect CSS, including age at diagnosis, histology, type of surgery, and adjuvant chemotherapy.

**TABLE 2:** Multivariable analysis of the influence of clinico‐pathologic factors on cancer‐specific survival among 1297 patients with early‐stage EOC.

Variables	Cancer‐specific survival		*p*
	HR	95% CI	
Age			
<35 years	1.00	Reference	
35–39 years	0.88	0.52–1.49	0.64
≥40 years	0.71	0.41–1.22	0.20
Histology			
Serous	1.00	Reference	
Mucinous	1.67	0.82–3.60	0.17
Endometrioid	0.94	0.46–2.02	0.87
Clear cell	0.81	0.41–1.70	0.55
Others^a^	0.72	0.25–1.87	0.51
Stage			
IA + IB	1.00	Reference	
IC	2.40	1.47–4.05	**<0.001**
II	3.60	1.86–6.93	**<0.001**
Grade			
1 + 2	1.00	Reference	
3	3.19	1.79–5.65	**<0.001**
Unknown	1.25	0.64–2.33	0.50
Type of surgery			
RCS	1.00	Reference	
FSS	1.09	0.66–1.77	0.73
Adjuvant chemotherapy			
No	1.00	Reference	
Yes	1.03	0.59–1.88	0.92

Abbreviations: CI, confidence interval; EOC, epithelial ovarian cancer; FSS, fertility‐sparing surgery; HR, hazard ratio; NOS, not otherwise specified; RCS, radical comprehensive staging surgery.

^a^ Others included mixed cell adenocarcinoma; adenocarcinoma, NOS; and carcinoma, NOS.

### 3.5 Correlations of various factors with outcomes in early‐stage EOC of different histologic types

We further analyzed the factors that correlated with outcomes of ovarian cancer of different histologic types: serous carcinoma, mucinous carcinoma, endometrioid carcinoma, and clear cell carcinoma. Table 3 shows the multivariable analyses of various clinico‐pathologic factors with regards to CSS in cases of four histologic types. Among patients with serous carcinoma, there was no obvious poor prognostic factor. In cases of mucinous carcinoma, poorer CSS was associated with Stage II (HR 7.44, 95% CI: 1.49–29.39, *p* = 0.007) or IC (HR 4.00, 95% CI: 1.55–11.11, *p* = 0.006) disease compared to Stage IA/IB disease, and with Grade 3 tumor (HR 3.33, 95% CI: 1.27–7.89, *p* = 0.009) compared to Grade 1/2 tumor. Among patients with endometrioid carcinoma, worse CSS was associated with Grade 3 tumor (HR 4.37, 95% CI: 1.46–12.89, *p* = 0.007) compared to Grade 1/2 tumor. In clear cell carcinoma, CSS was worse among patients with Stage II (HR 4.32, 95% CI: 1.24–14.40, *p* = 0.016) or IC (HR 3.08, 95% CI: 1.34–8.31, *p* = 0.014) disease compared to patients with Stage IA/IB disease. Additionally, among patients with clear cell carcinoma, CSS was better in patients who underwent FSS (HR: 0.28, 95% CI: 0.06–0.82, *p* = 0.040) than in patients who underwent RCS, and better in patients with adjuvant chemotherapy (HR: 0.32, 95% CI: 0.13–0.95, *p* = 0.020) compared to those without adjuvant chemotherapy.

**TABLE 3:** Multivariable analyses of the influence of various clinico‐pathologic factors on cancer‐specific survival among patients with early‐stage ovarian cancer, according to four histologic types.

	Serous (*N* = 123)	*p*	Mucinous (*N* = 365)	*p*	Endometrioid (*N* = 375)	*p*	Clear cell (*N* = 329)	*p*
	HR (95% CI)		HR (95% CI)		HR (95% CI)		HR (95% CI)	
Age								
<35 years	1.00 (Reference)		1.00 (Reference)		1.00 (Reference)		1.00 (Reference)	
35–39 years	1.07 (0.12–8.20)	0.95	1.07 (0.43–2.49)	0.88	0.99 (0.31–3.27)	0.99	0.75 (0.27–2.25)	0.58
≥40 years	0.70 (0.07–7.21)	0.76	0.65 (0.20–1.75)	0.42	0.76 (0.21–2.84)	0.67	0.73 (0.30–2.06)	0.52
Stage								
IA + IB	1.00 (Reference)		1.00 (Reference)		1.00 (Reference)		1.00 (Reference)	
IC	5.07 (0.77–101.58)	0.15	4.00 (1.55–11.11)	**0.006**	0.45 (0.15–1.37)	0.15	3.08 (1.34–8.31)	**0.014**
II	4.69 (0.52–109.19)	0.22	7.44 (1.49–29.39)	**0.007**	1.17 (0.31–4.14)	0.81	4.32 (1.24–14.40)	**0.016**
Grade								
1 + 2	1.00 (Reference)		1.00 (Reference)		1.00 (Reference)			
3	4.91 (1.00–36.33)	0.07	3.33 (1.27–7.89)	**0.009**	4.37 (1.46–12.86)	**0.007**	N/A	N/A
Unknown	1.92 (0.22–16.82)	0.53	0.89 (0.33–2.015)	0.80	2.33 (0.63–7.04)	0.16		
Type of surgery								
RCS	1.00 (Reference)		1.00 (Reference)		1.00 (Reference)		1.00 (Reference)	
FSS	2.02 (0.28–14.80)	0.48	1.76 (0.78–4.03)	0.17	2.27 (0.75–6.48)	0.13	0.28 (0.06–0.82)	**0.040**
Adjuvant C/T								
No	1.00 (Reference)		1.00 (Reference)		1.00 (Reference)		1.00 (Reference)	
Yes	3.14 (0.51–62.47)	0.31	1.12 (0.44–3.07)	0.81	1.81 (0.54–7.18)	0.36	0.32 (0.13–0.95)	**0.020**

Abbreviations: CI, confidence interval; C/T, chemotherapy; FSS, fertility‐sparing surgery; HR, hazard ratio; N/A, not available; RCS, radical comprehensive staging surgery.

### 3.6 Second malignancies among women with early‐stage EOC who underwent FSS

Of the 401 EOC patients who underwent FSS, 22 developed a second malignancy. As shown in Table 4, a second malignancy was defined as any type of cancer that was diagnosed more than 3 months after the diagnosis of EOC. The most common second malignancy was uterine endometrioid adenocarcinoma (*n* = 14), followed by ovarian cancer over the contralateral ovary (*n* = 3). Among all histological types, patients with endometrioid ovarian cancer had the highest frequency of developing second malignancies (8 out of 75, 10.7%).

**TABLE 4:** Patients with second malignancy in FSS group (*N* = 22).

Histology of EOC	Second malignancy	Total cases
Serous		1
	Colon	1
Mucinous		8
	Uterine corpus	4
	Ovary	1
	Colon	2
	Breast	1
Endometrioid		8
	Uterine corpus	7
	Ovary	1
Clear cell		3
	Uterine corpus	2
	Retroperitoneum	1
Others		2
	Uterine corpus	1
	Ovary	1

Abbreviations: EOC, epithelial ovarian cancer; FSS, fertility‐sparing surgery.

## 4 DISCUSSION

In this study, we used a nationwide registry to evaluate the outcomes of early‐stage EOC patients who underwent FSS and RCS. Patients in the FSS group were younger and mostly had Stage I disease. The RCS group included more cases of Stage II and high‐grade (Grade 3) disease, and more frequent adjuvant chemotherapy. The most common histologic type was mucinous carcinoma in the FSS group, compared to endometrioid and clear cell carcinomas in the RCS group. Stage was a risk factor for poor outcome for mucinous and clear cell histologies, but not for serous or endometrioid histology. Patients with Grade 3 endometrioid ovarian cancer had a poorer prognosis compared to patients with Grade 1/2 tumors. Among patients with early‐stage clear cell carcinoma, CSS was non‐inferior and even better after FSS compared to RCS, yet adjuvant chemotherapy was necessary. We also found that 22 out of the 401 women who underwent FSS developed a second malignancy later in life.

Several previous retrospective studies have compared the oncologic outcomes of early‐stage EOC patients who undergo FSS or RCS, and have reported comparable results.^10^, ^11^, ^12^, ^13^, ^14^, ^15^, ^16^, ^17^ The majority of these prior studies have only enrolled patients with Stage I disease, and have found that FSS is adequate treatment for Stage I EOC, without compromising survival outcomes.^11^, ^13^, ^14^, ^15^, ^16^ Ditto et al.^12^ and Bogani et al.^17^ analyzed the outcomes of FSS in patients with Stage I disease, and small numbers of patients with Stage II and III diseases. They reported that FSS did not influence the progression‐free survival (PFS) compared to complete staging surgery, among women with high‐risk ovarian cancer, with International Federation of Gynecology and Obstetrics (FIGO) Stage IA/IB Grade 3 or Stage IC/II diseases.^12^ Bogani et al. also reported that the type of surgery did not affect the disease‐free survival or overall survival (OS) of patients with Grade 3 tumors or Stage IC/II diseases after over 10 years of follow‐up.^17^ Similar to these past studies, in our present series, we found that FSS can be a safe procedure for patients with early‐stage EOC who had a desire for fertility preservation (FSS vs. RCS, HR of CSS 1.09, *p* = 0.73, Table 2).

We found that disease stage was an independent risk factor affecting outcome in patients with early‐stage EOC. Our previous results showed worse 5‐year OS in Stage II disease compared to Stage IA/IB disease.^4^ Ditto et al. also reported poorer OS in Stage IC/II diseases than Stage IA/IB diseases.^12^ In the present study, we found that Stage IC and II diseases were poor prognostic factors for CSS, with HR values of 2.40 and 3.60 relative to Stage IA/IB diseases. We further demonstrated that stage was an independent risk factor for poor CSS in cases of mucinous and clear cell histologies, but not cases of the serous or endometrioid type. Kajiyama et al. also reported that Stage IC disease was associated with poorer OS than Stage IA/IB disease in cases of the mucinous carcinoma and clear cell carcinoma histologies.^20^, ^21^ This indicates that the capsule status during operation significantly influences the outcomes, especially in mucinous and clear cell carcinomas.^20^, ^21^ Therefore, we recommend that surgeons should remove ovarian tumors as carefully as possible to avoid intraoperative tumor rupture, especially for patients undergoing FSS.

Tumor grade was another risk factor for poor outcome in early‐stage EOC patients. In our study, Grade 3 tumors were associated with worse CSS than Grade 1/2 tumors, especially in mucinous and endometrioid types **(**Table 3 **)**. Chen et al. also found that Grade 3 tumors were a poor prognostic factor compared to Grade 1/2 in cases of ovarian endometrioid carcinoma.^22^ Among cases of Stage I endometrioid carcinoma, Chao at el. also found that Grade 3 tumors were an independent poor prognostic factor in terms of PFS.^23^ However, it remains controversial whether grade correlates with unfavorable survival outcomes in mucinous carcinomas.^24^ Moreover, the grading system for ovarian mucinous carcinomatosis globally inconsistent.^24^ Busca et al. compared two widely used grading systems: the FIGO system,^25^ which was the same grading system used for endometrioid carcinoma, and the Silverberg grading system.^26^ They found that only the Silverberg grading system appeared to correlate with outcome in cases of mucinous carcinoma.^24^ In Taiwan, there is not yet a unified grading system used for mucinous ovarian carcinomas. Therefore, further investigation is needed to clarify how tumor grade influences mucinous carcinoma outcome.

**FIGURE 3:**
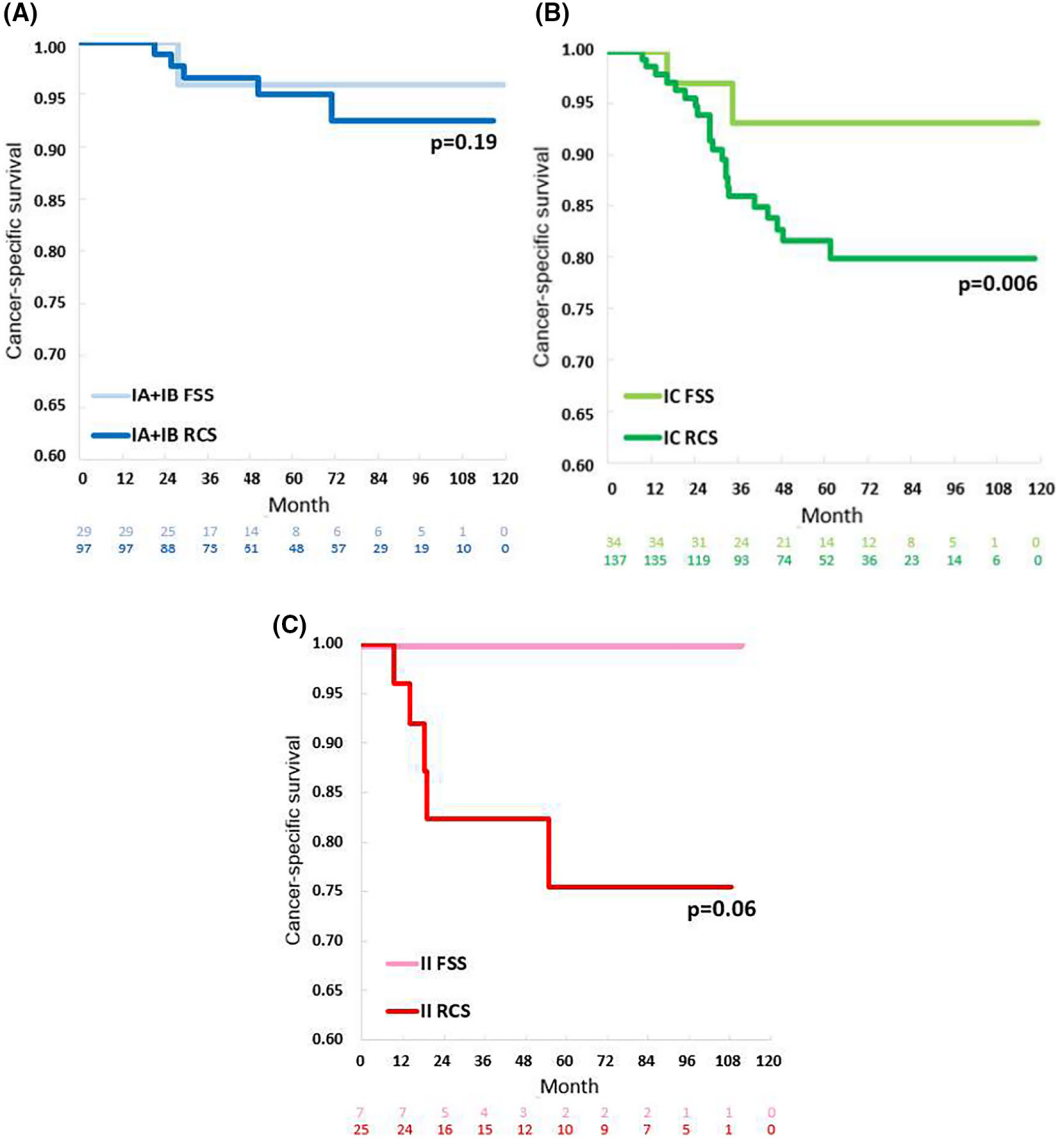
Kaplan–Meier analyses of cancer‐specific survival among patients with clear cell carcinoma stratified according to different type of surgical procedures in various stages: (A) Stages IA/IB, (B) Stage IC, and (C) Stage II.

There is a long‐standing debate regarding the safety of using FSS to treat patients with early‐stage ovarian cancer, especially clear cell carcinoma. Clear cell histology was considered a counterindication for FSS due to its relatively worse outcome compared to other histologic types.^27^ However, many recent retrospective studies have compared the outcomes of FSS and RCS for Stage I clear cell carcinoma, and the results reveal non‐inferior survival outcomes following FSS compared to RCS, although with limited patient numbers.^21^, ^28^, ^29^, ^30^, ^31^ Nasioudis et al. used the National Cancer Institute's Surveillance, Epidemiology, and End Results (SEER) database to evaluate cases of Stage I clear cell carcinoma treated with uterus‐ or ovary‐preserving staging surgery.^32^ Their study included a total of 741 patients with ovarian clear cell carcinoma, including 96 with uterus preservation. The 5‐year CSS rates did not significantly differ between patients with or without uterus preservation (90.8% vs. 87.7%, *p* = 0.29).^32^ Moreover, uterine preservation was not associated with worse survival even after controlling for the disease sub‐stage.^32^ However, in their review article, Satoh and Yoshikawa reported a higher cumulative relapse rate for patients with Stage IC clear cell carcinoma (22.6%) compared to patients with Stage IA clear cell carcinoma (11.1%).^33^ Thus, they did not recommend FSS for Stage IC clear cell carcinoma,^33^ and this indication remains controversial. Our present study included a large series obtained from the nationwide database, and our results showed that patients who underwent FSS for early‐stage clear cell carcinoma had survival outcomes similar to those of patients treated without fertility preservation. Within our nationwide database, the patients of reproductive age who underwent FSS were significantly younger than the patients who underwent RCS; however, our results showed no effect of age. Notably, among clear cell carcinoma patients, the FSS group even showed a significantly better survival outcome than the RCS group (HR 0.28, 95% CI: 0.06–0.82, *p* = 0.040). Further subgroup analysis revealed that the FSS and RCS groups had similarly good CSS rates in Stage IA/IB (Figure 3A, *p* = 0.19) and Stage II (Figure 3C, *p* = 0.06) clear cell carcinomas. In contrast, among Stage IC clear cell carcinomas, the FSS group had better CSS than the RCS group (Figure 3B, *p* = 0.006). A likely explanation is that the FSS patients may have been selected by surgeons during surgery, rather than randomly selected. Surgeons may choose the most suitable and low‐risk patients to undergo FSS, such as patients with an intact capsule and with minimal pelvic adhesion, to avoid potential spreading of cancer during surgery. We suggest that clear cell carcinoma patients who undergo FSS must receive regular and close surveillance following treatment.

Patients who undergo FSS might develop second malignancies in the uterine corpus or the contralateral ovary. In our study, ovarian endometrioid carcinoma was the histology most likely to develop second malignancies. Of the eight second malignancies in cases of ovarian endometrioid cancer, seven were uterine cancer. These cases illustrated the risk of uterus preservation with this histologic type. It is difficult to distinguish whether the second malignancy was a metastatic endometrioid endometrial cancer from the ovary or a synchronous cancer. Zhao et al. reported that patients with Stage I ovarian endometrioid carcinoma had a 19.3% rate of synchronous early stage and well‐to‐moderate differentiated endometrial carcinoma.^23^ Notably, in our study, uterine cancer also developed in patients with ovarian cancer of the other histologies, and all of these 14 uterine cancer were endometrioid endometrial cancer. Three patients developed ovarian cancer in the contralateral ovary. During surgery, biopsy of the contralateral ovary was usually not recommended in the absence of gross abnormalities, based on the low risk of microscopic involvement in a contralateral ovary with normal appearance,^14^ and concerns about infertility caused by postoperative adhesions on the remaining ovary.^34^, ^35^ However, patients should be informed that there is a risk of recurrence in the preserved contralateral ovary. Bentivegna et al. reported an 11.6% recurrence rate following FSS in patients with Stage I–II diseases. Among these recurrences, 38% were isolated in the spared ovary, and 62% occurred at an extraovarian site, which was associated with a worse survival outcome.^36^ Based on these findings, we suggest regular postoperative surveillance of the remaining ovary and of the endometrium using sonography or computerized tomography. Additionally, we recommend the performance of endometrial biopsy before or during FSS to identify synchronous endometrial and ovarian carcinomas, particularly in patients with ovarian endometrioid carcinoma with clinical symptoms, such as abnormal vaginal bleeding.

The present study had several strengths. The first strength was that it was a nationwide population‐based study performed using the TCR database, which includes over 90% of cancer patients in Taiwan.^19^ The database is periodically subjected to field data audits, and is thus a high‐quality and reliable data source.^19^ Additionally, this study has a large sample size, providing sufficient statistical power. Due to ethical problems, it is almost impossible to perform a prospective study comparing the outcomes of FSS and RCS^13^; therefore, we think that our study provides important new insights on this issue. Taiwan exhibits a higher incidence of clear cell carcinoma than many other countries.^37^ Thus, this study was able to include a large number of ovarian clear cell carcinoma patients, enabling comparison of outcomes after FSS or RCS specifically among ovarian clear cell carcinoma patients. We used CSS as the main outcome measure, instead of OS, because the proportion of death from other causes is relatively high in patients with early‐stage cancers.^38^ Therefore, CSS more precisely reflects the survival outcome related to early‐stage ovarian cancer.

One limitation of this study was its retrospective nature. There may have been some unavoidable difficulties in the coding and grouping of the patients, such as an inaccurate diagnosis or cancer staging, and loss of patients to follow‐up, which may lead to systemic bias. We lacked details of the pathologic findings, information regarding tumor recurrence, chemotherapy regimen and dosage, and the subsequent pregnancy records due to the nature of the TCR system. Tumor recurrence for each histology of EOC might provide more information to patient selection of FSS and adequate adjuvant therapy, and reflect the tumor nature of each histology. For instance, in high‐grade serous carcinoma, less cycles of adjuvant chemotherapy (three rather than six cycles) brought higher recurrence rate^39^; in mucinous carcinoma, traditional chemotherapy regimen brought higher recurrence rate than gastrointestinal type chemotherapy with or without bevacizumab^40^; in clear cell carcinoma, stage was a strong risk factor of recurrence^41^; in endometrioid carcinoma, Grade 3 tumor brought higher recurrence rate.^23^

The evolution of cancer treatment is an important, unmentioned limitation of this study. The preferable regimen of adjuvant chemotherapy changed from platinum (either carboplatin or cisplatin) and cyclophosphamide to platinum and paclitaxel in recent years, which may influence patients' oncologic outcome.^42^ Although a trend of delayed child birth does exist in Taiwan (average age of first childbirth: 29.28 to 30.83 y/o, 2009–2017), the proportion of FSS does not significantly increase in these years. It may be attributed to the controversial selection criteria of FSS and the importance of this study.

In summary, our present results showed that FSS can be a safe alternative treatment in selected young patients with Stage I ovarian cancer and a wish for fertility preservation. A detailed presurgical consultation should be offered. Additionally, patients treated with FSS must receive regular and close surveillance, including endometrial biopsy and gynecologic sonography, to detect early disease recurrence after treatment.

## AUTHOR CONTRIBUTIONS

**Chia‐Yi Lee:** Data curation (supporting); formal analysis (supporting); methodology (supporting); writing – review and editing (supporting). **Chun‐Ju Chiang:** Data curation (equal); formal analysis (supporting); methodology (supporting); writing – review and editing (equal). **Yi‐Jou Tai:** Formal analysis (supporting); methodology (supporting); writing – review and editing (supporting). **Heng‐Cheng Hsu:** Data curation (supporting); formal analysis (supporting); methodology (supporting); writing – review and editing (supporting). **Yu‐Li Chen:** Formal analysis (supporting); methodology (supporting); writing – review and editing (supporting). **Ying‐Cheng Chiang:** Formal analysis (supporting); methodology (supporting); writing – review and editing (supporting). **Chia‐Ying Wu:** Formal analysis (supporting); writing – review and editing (supporting). **Wen‐Chung Lee:** Conceptualization (supporting); funding acquisition (lead); project administration (lead); supervision (lead); writing – review and editing (supporting). **Hsiao‐Lin Hwa:** Methodology (supporting); writing – review and editing (supporting). **Wen‐Fang Cheng:** Conceptualization (equal); project administration (supporting); supervision (equal); writing – original draft (supporting).

## FUNDING INFORMATION

This study was funded by the Health Promotion Administration, Ministry of Health and Welfare, grant no. A1091115: Tobacco Health and Welfare Taxation. The content of this research may not represent the opinion of the Health Promotion Administration and Ministry of Health and Welfare. The funders had no role in study design, data collection and analysis, the decision to publish, or preparation of the manuscript.

## CONFLICT OF INTEREST STATEMENT

No potential conflicts of interest were disclosed.

## ETHICS STATEMENT

This study was approved by the institutional Research Ethics Committee at the National Taiwan University Hospital (approval No. 201907088RIN). All of the patients' data were fully anonymized before we accessed them and the Research Ethics Committee waived the requirement for informed consent.

## Supporting information


Table S1.

## Data Availability

The data that support the findings of this study are available on request from the corresponding author. The data are not publicly available due to privacy or ethical restrictions.
