# Supplementary material for: Outcomes after fertility‐sparing surgery of early‐stage ovarian cancer: A nationwide population‐based study
Source: Cancer Med. 2024 Apr 12;13(7):e7132. doi: 10.1002/cam4.7132 (PMC11010646; doi:10.1002/cam4.7132)
Supplement: Supplementary file 1 — Table S1. [file CAM4-13-e7132-s001.docx]

**Supplementary Table 1.**Basic characteristics of 1297 early-stage EOC patients with or without adjuvant chemotherapy

| **Characteristics** | **Adjuvant chemotherapy (N=913)  (%)** | **Without adjuvant chemotherapy  (N= 384)  (%)** | ***p*** |
| --- | --- | --- | --- |
| **Age, median (range)** | 39 (20–44) | 36 (20–44) | **<.0001** |
| **Age**  <35 years  35–39 years  ≥40 years | 240 (26.3)  266 (29.1)  407 (44.6) | 166 (43.2)  94 (24.5)  124 (32.3) | **<.0001** |
| **Histology**  Serous carcinoma  Mucinous carcinoma  Endometrioid carcinoma  Clear cell carcinoma  Others^*^ | 83 (9.1)  176 (19.3)  280 (30.6)  302 (33.1)  72 (7.9) | 40 (10.4)  189 (49.2)  95 (24.8)  27 (7.0)  33 (8.6) | **<.0001** |
| **Stage**  IA+IB  IC  II | 261 (28.6)  513 (56.2)  139 (15.2) | 316 (82.3)  63 (16.4)  5 (1.3) | **<.0001** |
| **Grade**  1+2  3  Unknown | 358 (39.2)  443 (48.5)  112 (12.3) | 222 (57.8)  48 (12.5)  114 (29.7) | **<.0001** |
| **Surgical procedure**  FSS  RCS | 215 (23.6)  698 (76.4) | 186 (48.4)  198 (51.6) | **<.0001** |

*EOC* epithelial ovarian cancer, *FSS* fertility-sparing surgery, *RCS* radical comprehensive staging surgery, *N* number of patients. *Others included mixed cell adenocarcinoma; adenocarcinoma, not otherwise specified (NOS); and carcinoma, NOS.
